# Drexel University’s Age-Friendly Strategy: Starting With Purposeful and Existing Engagement Mechanisms

**DOI:** 10.1093/geroni/igaa057.1807

**Published:** 2020-12-16

**Authors:** Katherine Clark, Laura Gitlin, Rose Ann DiMaria-Ghalili, Elizabeth Yutzey

**Affiliations:** 1 Drexel University, Philadelphia, Pennsylvania, United States; 2 Drexel University, Jenkintown, Pennsylvania, United States; 3 Drexel University, College of Nursing and Health Professions, Philadelphia, Pennsylvania, United States

## Abstract

In 2019, Drexel University became the first Age-Friendly University (AFU) in Philadelphia, Pennsylvania. The College of Nursing and Health Profession’s AgeWell Collaboratory (a center without walls that aims to disrupt the healthcare system’s traditional approach to aging) is leading the Age-friendly Drexel Steering Committee, which is composed of leadership throughout the university. The Collaboratory purposefully connected the committee to four key mission-driven efforts in order to ensure sustainability: 1)Strategic planning, both at the university and college level 2)The institution’s research agenda 3)Existing programming and work groups (ie. The University Committee on Accessibility and the School of Professional Development and Institutional Advancement’s efforts to engage more older adults),4) Community engagement efforts, such as the City of Philadelphia’s Age-friendly initiative and the university’s community outreach hub, the Dornsife Center for Neighborhood Partnerships. This presentation will discuss how to leverage the AFU movement through mission-driven efforts among senior leadership, faculty, staff and students.

